# Comparison of the protective effectiveness of NPQ in *Arabidopsis* plants deficient in PsbS protein and zeaxanthin

**DOI:** 10.1093/jxb/eru477

**Published:** 2014-11-26

**Authors:** Maxwell A. Ware, Erica Belgio, Alexander V. Ruban

**Affiliations:** School of Biological and Chemical Sciences, Queen Mary University of London, Mile End Road, London E1 4NS, UK

**Keywords:** *Arabidopsis*, protective NPQ, photoinhibition, photosystem II, PsbS protein, zeaxanthin.

## Abstract

The efficiency of protective energy dissipation by non-photochemical quenching (NPQ) in photosystem II (PSII) has been recently quantified by a new non-invasive photochemical quenching parameter, qPd. PSII yield (ФPSII) was expressed in terms of NPQ, and the extent of damage to the reaction centres (RCIIs) was calculated via qPd as: ФPSII=qPd×(*F*_v_/*F*_m_)/{1+[1–(*F*_v_/*F*_m_)]×NPQ}. Here this approach was used to determine the amount of NPQ required to protect all PSII reaction centres (pNPQ) under a gradually increasing light intensity, in the zeaxanthin-deficient (*npq1*) *Arabidopsis* mutant, compared with PsbS protein-deficient (*npq4*) and wild-type plants. The relationship between maximum pNPQ and tolerated light intensity for all plant genotypes followed similar trends. These results suggest that under a gradually increasing light intensity, where pNPQ is allowed to develop, it is only the amplitude of pNPQ which is the determining factor for protection. However, the use of a sudden constant high light exposure routine revealed that the presence of PsbS, not zeaxanthin, offered better protection for PSII. This was attributed to a slower development of pNPQ in plants lacking PsbS in comparison with plants that lacked zeaxanthin. This research adds further support to the value of pNPQ and qPd as effective parameters for assessing NPQ effectiveness in different types of plants.

## Highlight

A novel method for assessing the protective effectiveness of non-photochemical chlorophyll fluorescence quenching revealed that PsbS protein plays a generally more important role then zeaxanthin.

## Introduction

Since the divergence of land plants between 400 million years ago (Mya) and 700 Mya (Heckman *et al.*, 2001; Sanderson, 2003), they have had to overcome gravity, dehydration, and significant fluctuations in light intensity (Raven, 1984, 1994; Niklas, 1998; Ruban, 2012). The latter can occur due to temporal and spatial differences in light environments, such as seasonal variations in sunlight and scattering due to cloud movement, respectively. High light exposure can lead to photodamage in the crucial but vulnerable component of the photosynthetic machinery, the photosystem II (PSII) reaction centre (RCII) (Powles, 1984; Aro *et al.*, 1993*b*; Barber, 1995). PSII is composed of major light-harvesting complex II (LHCII) and minor (CP24, CP26, and CP29) light-harvesting antenna proteins, core antenna complexes (CP43 and CP47), and the RCII core complex (Bassi *et al.*, 1987; Boekema *et al.*, 1995; Andersson *et al.*, 2001; Dekker and Boekema, 2003; Yakushevska *et al.*, 2003). Responsible for the generation of electrons that initiate the photosynthetic electron transport reactions and evolution of oxygen, PSII is fundamental to all life on our planet (Melis, 1999; Barber, 2002, 2009; Umena *et al.*, 2011). The LHCII complexes bind chlorophyll *a*, chlorophyll *b*, and a variety of oxygenated carotenoids, and xanthophylls, and function as a major source of increase in the energy input into RCII (Peter and Thornber, 1991; Bassi and Dainese, 1992; Bassi *et al.*, 1993; Ruban, 2012). Energy absorption and transfer from the antenna to RCII is much faster than the subsequent electron transfer. Hence, under increasing light intensities, there is a growing disparity between light absorption and the energy which is used for photochemical work (Björkman and Demmig, 1987; Adams *et al.*, 2006; Demmig-Adams *et al.*, 2012; Ruban, 2012). At high light intensities there is a build-up of excess energy in PSII, which via the generation of reactive oxygen intermediates can cause photoinhibition, manifesting in the long-term closure and damage to RCII (Ohad *et al.*, 1984; Aro *et al*., 1993*a*; Barber, 1995). This has led to the physiological requirement for a way of preventing the accumulation of excess energy in PSII.

Non-photochemical quenching of chlorophyll fluorescence (NPQ) is a process that serves to counteract the build-up of excess energy in PSII, by dissipating the excitation energy into heat (Briantais *et al.*, 1979; Horton and Ruban, 1992; Müller *et al.*, 2001; Jahns and Holzwarth, 2012; Ruban *et al.*, 2012). NPQ is known to consist of several components: qE, qT, qZ, and qI, recognized by the rate of their formation and relaxation (Adams *et al.*, 2006; Jahns and Holzwarth, 2012; Ruban *et al.*, 2012). The fastest and most clearly understood component is qE, or energy-dependent quenching, that is triggered by the generation of a pH gradient (ΔpH) across the thylakoid membrane (Krause and Behrend, 1988; Krause *et al.*, 1988; Noctor *et al.*, 1991; Horton and Ruban, 1992). qT, indicative of state transitions, balances energy absorption at low light between PSII and photosystem I (PSI) (Krause and Weis, 1991; Allen and Fornsberg, 2001; Ruban and Johnson, 2009). qZ is zeaxanthin-dependent NPQ (Nilkens *et al.* 2010; Brooks *et al.*, 2013), and qI is quenching associated with closed RCIIs due to photoinhibition (Krause, 1988; Jahns and Holzwarth, 2012; Ruban *et al.*, 2012).

qE formation has been detected as heat emission in 1.4 μs and relaxes in minutes (Krause and Weis, 1991; Horton and Ruban, 1992; Mullineaux *et al.*, 1994; Johnson *et al.*, 2009). Apart from ΔpH, two additional key factors are now widely accepted to be involved in the formation of qE: the antenna carotenoid zeaxanthin and the PsbS protein. Although required for ATP production during the light-dependent stages of photosynthesis, at high light, ΔpH not only triggers qE (Wraight and Crofts, 1970; Krause, 1974; Horton *et al.*, 2005) but also results in the de-epoxidation of violaxanthin into zeaxanthin (Sapozhnikov *et al.*, 1957; Demmig *et al.*, 1987; Demmig-Adams, 1990; Bugos *et al.*, 1996, Müller *et al.*, 2001). Acidification of the thylakoid lumen also leads to the protonation of lumen-exposed amino acid residues of the PsbS protein (Funk *et al.*, 1995; Li *et al.*, 2000; Li *et al.*, 2002*b*). It was proposed that zeaxanthin promotes NPQ by acting as an allosteric modulator in the PSII membrane, enhancing LHCII–proton binding affinity, thus activating qE at a higher lumenal pH than in *npq1* mutants (Ruban and Horton, 1999; Ruban, 2012, Ruban *et al.*, 2012). Li *et al.* (2002*a*) showed that there was a positive correlation between the amount of PsbS protein and the maximum capacity for qE in *Arabidopsis* plants, and it has been subsequently shown that PsbS protein affects membrane rigidity. Goral *et al.* (2012) showed that plants lacking PsbS have reduced mobility of grana membrane proteins, whilst Kereïche *et al*. (2010) demonstrated increased incidence of semi-crystalline ordered arrays of PSII supercomplexes in *npq4* plants. Both zeaxanthin (Demmig-Adams, 1990; Dreuw *et al.*, 2003) and the PsbS protein (Li *et al.*, 2000; Müller *et al.*, 2001) have been previously thought to be essential for qE. However, it has subsequently been shown that qE can form in knock-out mutants of either violaxanthin de-epoxidase (Rees *et al.*, 1989; Niyogi *et al.*, 1997) or PsbS protein (Johnson and Ruban, 2011), the latter being in the presence of diaminodurene. Despite this, the molecular mechanism of the energy conversion into heat underlying qE has so far remained a subject of intense debates (Jahns and Holzwarth, 2012; Ruban *et al.*, 2012).

The more slowly reversible component of NPQ is generated as a result of photoinhibition in RCII and is termed qI (Krause, 1988; Jahns and Holzwarth, 2012). Simultaneous assessment of the protective components of NPQ (qE, qZ, and qT) and photoinhibition (qI) has proved difficult as estimation of qI-related RCII damage requires invasive techniques, such as the tracking of D1 protein degradation (Greenberg *et al.*, 1987; Aro *et al.*, 1993*a*; Tyystjärvi and Aro, 1996). Monitoring fluorescence offers a simple way of observing the onset of NPQ as it results in a decline in the yield of chlorophyll fluorescence (Wraight and Crofts, 1970; Briantais *et al.*, 1979; Schreiber, 1986; Genty *et al.*, 1989). Here it is shown that fluorescence quenching analysis utilizing a gradually increasing actinic light routine can allow the measurement of NPQ and the quantification of the protective component, pNPQ, on whole intact leaves. Furthermore, this measurement can be performed regardless of the rates of NPQ formation or relaxation, which in itself is an often ambiguous criterion, or the subsequent repair mechanisms of PSII. Another benefit of this technique is that it allows the maximum NPQ to be formed in plants without the rate of NPQ formation being a selection criterion. Using this technique, the onset of qI can also be measured as a decline in the quantum coefficient of photochemical quenching in the dark immediately after illumination (qPd) or as the quantum yield of PSII (ΦPSII). This allows calculation of the maximum tolerated light intensity at which 100% of RCs remain undamaged, 50% RC damage, and the minimum light intensity which induces 100% RC damage.

Using a slightly different approach, the rates and amplitude of qPd decline and NPQ formation were also quantified by exposing plants to a constant high actinic light intensity. This routine was repeated with the uncoupler nigericin. It has been previously shown that nigericin, by dissipating ΔpH and membrane energization, can be used to distinguish between reversible, ΔpH-dependent, and irreversible inhibitory fluorescence quenching (Neubauer and Yamamoto, 1992; Neubauer, 1993; Ruban and Murchie, 2012). Running the same routine on leaf samples, with and without nigericin, allowed the calculation of NPQ attributed to qI. This constant high light routine shows that in order to measure pNPQ effectively, a gradually increasing light routine must be used to allow NPQ–actinic light intensity equilibrium to be established. The constant high light routine therefore allowed for quantitative comparison between PsbS protein and zeaxanthin, when pNPQ is not given a chance to establish fully. This allows a comparison between the rates of NPQ formation and qPd decline when speed as well as the quantity of NPQ is important. The current work quantified the ‘protectiveness’ of NPQ generated by the PsbS protein and zeaxanthin. Using this technique, these two vital molecules can be compared to see which one confers more efficient photoprotection.

## Materials and methods

### Plant material

Colombia-0, wild type (WT), *Arabidopsis thaliana* ecotype, *npq1* (unable to convert violaxanthin to zeaxanthin), and *npq4* (lacking PsbS protein) were grown on a ratio of 6:6:1 Levington M3 potting compost, John Innes No. 3 soil, and perlite (Scotts UK, Ipswich, UK). Seeds were sterilized by soaking in 2ml Eppendorf tubes with 50% ethanol and 0.1% Triton X-100 for 3min before washing three times in distilled water and subjecting them to cold treatment (4 °C) for 3 d. Plants were raised under 58W Linear Fluorescent Tube Cool White with an 8h photoperiod at 175 μmol m^–2^ s^–1^ light and 21 °C. All plants were subjected to 45min dark adaptation before each measurement. Measurements were performed on whole plants with intact leaves, aged between 40 d and 60 d after sowing. Where indicated, detached leaves were vacuum infiltrated with 100 μM nigericin and 20 μM HEPES (pH 7.0) after dark adaptation.

### Fluorescence measurements

**Fig. 1.**
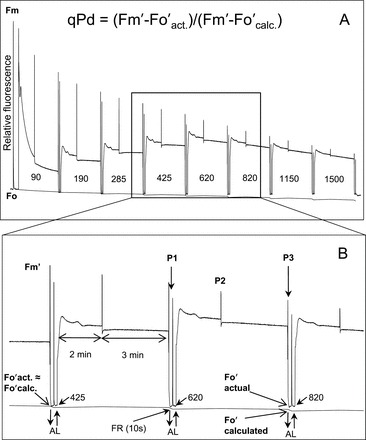
(A) Scheme of induction of chlorophyll fluorescence from an *npq4* plant with an eight step increasing actinic light (AL) routine. In this example, 0, 90, 190, 280, 420, 625, 820, 1150, and 1500 μmol m^–2^ s^–1^ AL intensities were used. In order to increase the range of data and attempt to reflect leaf variation accurately, three other intensity ranges were used in other experiments, being either 80, 87, or 90% of the aforementioned values. These intensities were achieved by carefully withdrawing the fibre-optic from the Junior-PAM (Walz) emitting diode and measuring the AL intensity with a Walz MQS-B sensor. For detailed explanation of routine development, see Ruban and Belgio (2014). (B) Inset illustrating the timing and application of AL (upward arrow and downward arrows demonstrate the turning of AL on and off, respectively), along with saturating pulses (SPs) (P1, P2, and P3). P1 illustrates an SP before actinic light illumination, P2 during AL illumination, and P3 at the end of AL exposure and prior to 10 s of far red light (FR). The difference between actual and calculated *F*_o_′ used to calculate qPd is also shown. At low AL intensities, *F*_o_ʹ calc. and *F*_o′_ act. match or are extremely close, but, under increasingly high irradiance, the two values diverge. See also the Materials and methods for a detailed description. The timing scheme of the qPd calculation and darkness step of the routine was: (AL off) (FR on)–(10 s)–(SP)–(5 s)–(AL on/FR off).

Fluorescence traces were recorded using a Walz JUNIOR PAM fluorometer (Walz Effeltrich, Germany), fluorescence standard foil and monitoring leaf clip. The quenching run, previously described in Ruban and Belgio (2014), lasted ~42min and is illustrated in Fig. 1. Eight different actinic light intensities were used in one measurement. Light intensities for the gradually increasing actinic light routine were 0, 90, 190, 285, 420, 625, 820, 1150, and 1500 μmol m^–2^ s^–1^. A range of other intensities from 72 μmol m^–2^ s^–1^ to 1350 μmol m^–2^ s^–1^ were achieved by the gradual removal of the fibre-optic from the diode in the JUNIOR-PAM device, and confirmed by the Walz MQS-B sensor. A total of 40 repeats for each genotype were undertaken, with 10 at each of the four illumination sequences. For constant high light routines, eight successive actinic light illuminations of 1500 μmol m^–2^ s^–1^ were used. ΦPSII and qPd parameters were recorded at P1, and NPQ values were measured at P3 (Fig. 1).

### Theory

ΦPSII is affected by NPQ and RCII photodamage. It was therefore deemed of great importance to distinguish the ΦPSII reduction attributed to the quickly reversible NPQ, and the long-lasting photodamage. The following formula thus relates ΦPSII to NPQ, qPd, and *F*_v_/*F*_m_:

$$\Phi \text{PSII}=qPd\frac{\left(\frac{Fv}{Fm}\right)}{\left[1+\left(1-\frac{Fv}{Fm}\right)NPQ\right]}$$ (1)

where qPd is the photochemical quenching (qP) measured in the dark immediately following a period of illumination; *F*_m_ is the maximum fluorescence in the dark-adapted leaf; *F*_v_=*F*_m_–*F*_o_, where *F*_o_ is the dark fluorescence level before illumination and *F*_v_/*F*_m_ is the maximum quantum yield of PSII. In the absence of photoinhibition, qPd=1. Here the theoretical yield of PSII and the actual yield are extremely well matched. However, upon the onset of damaged reaction centres, qPd becomes <1, and the actual and theoretical yields diverge.

qPd was calculated using the previously described formula (Ruban and Murchie, 2012):

$$qPd=\frac{Fm^{\prime }-Fo^{\prime }\text{act.}}{Fm^{\prime }-Fo^{\prime }\text{calc.}}$$ (2)

where *F*_m_′ is the maximum fluorescence after actinic light illumination; *F*_o_′_act_ is the measured dark level of fluorescence after illumination, and *F*_o_′_calc_ is the calculated dark fluorescence level. The latter was calculated using the formula proposed by Oxborough and Baker (1997):

$$Fo^{\prime }\text{calc.}=\frac{1}{\left(\frac{1}{Fo}-\frac{1}{Fm}+\frac{1}{Fm^{\prime }}\right)}$$ (3)

Displayed in Fig. 1, at low light intensities, the measured level of *F*_o_′ (here termed *F*_o_′_act._) and the *F*_o_′_calc._ are matched, but at high light intensities they deviate. Using these two values, qPd can be calculated.

Therefore. when used in concert, qPd and ΦPSII can be used to measure the onset of photoinhibition accurately. When qPd=1, 100% of RCs are open and being protected by NPQ. This has led to the terminology protective NPQ (pNPQ) being used to describe the amount of NPQ required to stop any onset of photoinhibition.

### Statistical analysis

Single-factor analysis of variance (ANOVA) was used to signify the difference between group means for different parameters (NPQ, qPd, and pNPQ). It is a method of comparing whether means are equal for more than two groups. *P*<0.05 was used as the significant value for all ANOVA tests, which can be confirmed by *F*-value >*F* critical value.

Student’s *t*-tests were used to test statistically whether the difference between the mean of two groups was significant. A two-tailed distribution was used as values both side of the mean were considered equally likely. *P*<0.05 was used as the significant value for all *t*-tests. In all figures, where a *t*-test has been employed, a single asterisk is used to signify this significant difference between mutants and WT plants. A double asterisk represents a significant difference between *npq1* and *npq4* plants.

A *z*-test was employed to check whether the rate and amplitude were different between two sets of data. This test analyses one parameter (often the mean) and encompasses the standard error of the parameter. A two-tailed distribution was used as values both side of the mean were considered equally likely. *z*-tests have a critical value for all percentage confidence intervals. Here a 5% (therefore *P*=0.05) confidence interval was used for a two-tailed distribution *z*-test, so the critical value was 1.96. In all figures, where a *z*-test has been used, a single asterisk is used to signify this significant difference between mutants and WT plants. A double asterisk represents a significant difference between *npq1* and *npq4* plants.

Error bars represent either the standard deviation:

$$\text{SD}=\frac{\sqrt{\sum {\left(\text{x}-\overset{-}{\text{x}}\right)}^{2}}}{\text{n}}$$ (4)

or the standard error of the mean:

SEM=SD/√n (5)

Indications are given in the figure legends of the statistical analysis employed.

## Results

### Induction of NPQ using two different pulse amplitude modulated (PAM) fluorescence routines

Figure 1 shows a representative fluorescence trace recorded for one experiment conducted on an *npq4* leaf. The program-encoded scheme is: (SP)–(AL on)–(120 s)–(SP)–(180 s)–(SP)–(AL off/FR on)–(10 s)–(SP)–(5 s)–(AL on/FR off)–repeat. Here AL represents actinic light, SP the saturating pulse, and FR is far red light. Eight different actinic light intensities were used in each measurement. In order to increase the variations in actinic light intensity between measurements and collect a more representative spread of data, the distance of the fibre-optics from the light diode was adjusted a number of times (for a detailed description, see Ruban and Belgio, 2014). At the end of each actinic light illumination, *F*_o_′ was measured in the dark, thus representing the actual level of *F*_o_′ (*F*_o_′ act.). This value was then compared via the qPd parameter (see Equation 3 in the Materials and methods) with the theoretical *F*_o_′ level in the absence of photoinhibition (*F*_o_′ calc.), estimated from the Oxborough and Baker formula.

At low light intensities (see Fig. 1) there is little to no difference between the two *F*_o_ values. At high light, however, photoinhibition of RCIIs leads to an increase in *F*_o_′ act., resulting in a discrepancy between the two values (see Fig. 1 inset for the difference between *F*_o_′ act. and *F*_o_′ calc. when the actinic light of 820 μmol m^–2^ s^–1^ was switched off). Since the theoretical *F*_o_′ value is calculated purely in terms of *F*_m_ and *F*_m_′ (see Equation 3), the zoomed area indicates that *F*_o_′ act. was not appreciably quenched as was expected from the relative *F*_m_’ measured just before the actinic light was switched off. As a consequence, the qPd parameter at these light intensities dropped below 1. In order to account for the natural variations in qPd values, qPd <0.98 was selected as a mark of photoinhibition, meaning that below this value >2% of RCIIs are damaged and the damage is relatively proportional to the decrease of qPd. The 5min interval between actinic illumination increases (Fig. 1) was sufficient for the onset of pNPQ, as well as the establishment of a relatively steady level of photoinhibition at inhibitory light intensities (Ruban and Belgio, 2014).

In order to monitor the maximum rate of the onset of photoinhibition and pNPQ, another fluorescence induction routine has been used in this study (Supplementary Fig. S1 available at *JXB* online). Here the timing was kept identical to the routine described above with eight illumination cycles, but the actinic light intensity was set to 1500 μmol m^–2^ s^–1^ from the very beginning. The combination of the two procedures enabled a distinction to be made between the impacts of NPQ kinetics and its amplitude on RCII photoprotection in the two *Arabidopsis* mutants used in this study.

### Comparing the protective efficiency of NPQ in *npq1* and *npq4* plants under a gradually increasing actinic light routine

**Fig. 2.**
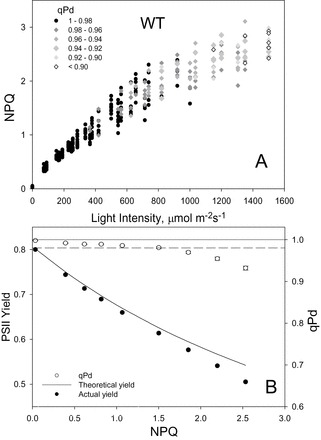
(A) Relationship between light intensity and NPQ, and the subsequent effect on qPd for 40 WT *Arabidopsis* intact leaves. Data points were taken during the fluorescence routine explained in Fig. 1. The figure key explains the greyscale relationship of symbols to qPd. (B) Relationship between NPQ, PSII actual yield (filled circles), and qPd (open circles). At each light intensity, NPQ and qPd data points were taken from A and averaged. Error bars show the SEM (*n*=40). The theoretical yield (continuous line) was calculated using Equation 1 of the Materials and methods.

**Fig. 3.**
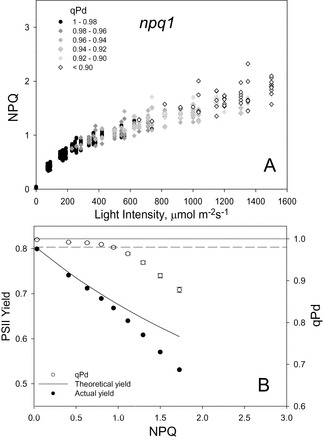
(A) Relationship between light intensity and NPQ, and the subsequent effect on qPd for 40 *npq1 Arabidopsis* intact leaves. Data points were taken during the fluorescence routine explained in Fig. 1. The figure key explains the greyscale relationship of symbols to qPd. (B) Relationship between NPQ, PSII actual yield (filled circles), and qPd (open circles). At each light intensity, NPQ and qPd data points were taken from A and averaged. Error bars show the SEM (*n*=40). The theoretical yield (continuous line) was calculated using Equation 1 of the Materials and methods.

**Fig. 4.**
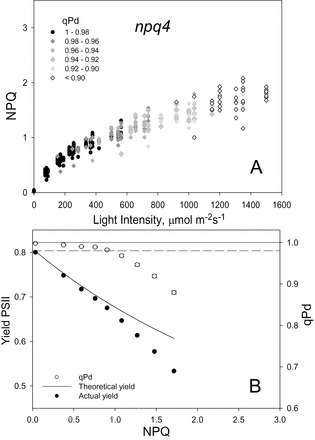
(A) Relationship between light intensity and NPQ, and the subsequent effect on qPd for 40 *npq4 Arabidopsis* intact leaves. Data points were taken during the fluorescence routine explained in Fig. 1. The figure key explains the greyscale relationship of symbols to qPd. (B) Relationship between NPQ, PSII actual yield (filed circles), and qPd (open circles). At each light intensity, NPQ and qPd data points were taken from A and averaged. Error bars show the SEM (*n*=40). The theoretical yield (continuous line) was calculated using Equation 1 of the Materials and methods.

Figures 2A–4A show the relationship between NPQ and actinic light intensity in WT, *npq1*, and *npq4* plants. At each light intensity, the calculated qPd is shown as a measure of photoinhibition. Black circles show the measurements which resulted in qPd values >0.98, therefore cases where negligible photoinhibition occurred, whilst for those cases where qPd went below 0.98, grey rhomboid symbols were used. This method enabled three-dimensional data sets to be presented effectively using simple two-dimensional plots. Whilst the WT plants were able to form a maximum NPQ of 3.1, in *npq1* and *npq4* mutants this parameter only reached 2.3 and 2.1, respectively. At up to ~400 μmol m^–2^ s^–1^, NPQ in all three genotypes had a similar amplitude; however, above this light intensity the mutants were incapable of forming much higher NPQ. ANOVA (*P*=1.5×10^–12^) confirmed a significant difference between the NPQ formed in the WT, *npq1*, and *npq4* genotypes. In particular, both mutants formed significantly less NPQ than the WT over the routine (*t*-test, *P*<0.001). There was not, however, a significant difference between *npq1* and *npq4* plants (*P*=0.47), thus statistically proving that mutants lacking zeaxanthin and PsbS protein form similar amounts of NPQ over the same gradually increasing actinic light routine. It is important to mention that the *t*-test comparisons of *F*_v_/*F*_m_ between all genotypes showed no significant difference between the WT, *npq1*, or *npq4* (*P*>0.5) and the mean *F*_v_/*F*_m_ for all genotypes was >0.8. Such a high PSII yield was the result of the improvement in plant growth conditions (see the Materials and methods) compared with 0.77 in the plants that were grown under very low light conditions used previously (Ruban and Belgio, 2014). It was clear from Ruban and Belgio (2014) that during the gradual routine, there was only an ~0.25 drop in ΦPSII. It was therefore of paramount importance to maximize plant health in order to be as accurate as possible.

It has previously been shown that deviation of ΦPSII from the theoretical relationship derived by Ruban and Murchie (2012) was another indication of the critical light intensity at which the onset of photoinhibition has occurred. At each light intensity, shown in the top panel of Figs 2–4, the data points were averaged and a relationship between NPQ and ΦPSII, and NPQ and qPd was therefore derived for the three genotypes in addition to the NPQ versus light intensity analysis (Figs 2B–4B). In both mutants, the onset of photoinhibition took place at a much lower amount of NPQ than in the WT, as the theoretical yield started to deviate from the actual yield much earlier. This simply suggests that they were unable to form NPQ of amplitude sufficient to counteract the detrimental effect of increased light intensity. Early signs of photoinhibition appeared later in the WT, when the actinic light induced damage on average with NPQ equal to ~1.5, whilst for *npq1* and *npq4* plants, photoinhibition is evident when NPQ is ~0.9. A significant difference was thus found between the genotypes for qPd (ANOVA, *P*=4.76×10^–9^), while *t*-tests showed that there was no statistical difference between the *npq1* and *npq4* mutants (*P*=0.44). This suggests that they are equally protected against photodamage, but less protected than WT plants (*npq1*, *P*=2.24×10^–9^; *npq4*, *P*=7.78×10^–8^).

**Fig. 5.**
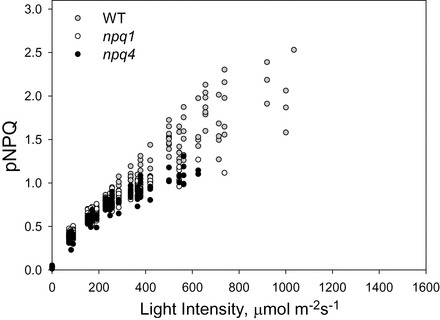
Relationship between protective NPQ (pNPQ) and actinic light intensity taken from all the data points in Figs 2A–4A where qPd >0.98. White circles represent *npq1* measurements, grey are WT, and black are *npq4*. All results appear on approximately the same gradient (see Supplementary Fig. S2A–C at *JXB* online). An NPQ value of 1 can protect 100% of reaction centres up to 575 μmol m^–2^ s^–1^ and a value of 2 up to 1050 μmol m^–2^ s^–1^ (taken from Supplementary Fig. S2A–C).

In order to compare the relationship between pNPQ and light intensity in the three genotypes, the data points from Figs 2A–4A corresponding to qPd >0.98 have been plotted in Fig. 5. A strong overlap between the WT, *npq1*, and *npq4* plants can be observed; however, the WT plants were able to endure higher light intensities without suffering damage. Indeed some WT plants were able to generate pNPQ up to ~2.5 compared with mutant plants generating up to ~1.3. The *t*-test analysis showed that the pNPQ values from *npq1* and *npq4* plants were not statistically different (*P*=0.99). From this plot, the data points corresponding to the minimum pNPQ required to protect RCIIs at a given light intensity were taken (Supplementary Fig. S2A–C at *JXB* online), showing that all genotypes followed a similar linear gradient (*z*-test, *P*>0.1). The straight line was in fact very similar in the three cases (see the relevant figure legends), suggesting that, regardless of the presence or absence of zeaxanthin or PsbS, the absolute amplitude of NPQ is the only factor governing photoprotection. Hence, pNPQ of 2.0 in any of the studied plants can confer protection up to a maximum of ~1050 μmol m^–2^ s^–1^ (Supplementary Fig. S2).

### Quantifying light tolerance of plants under a gradually increasing actinic light intensity routine

**Fig. 6.**
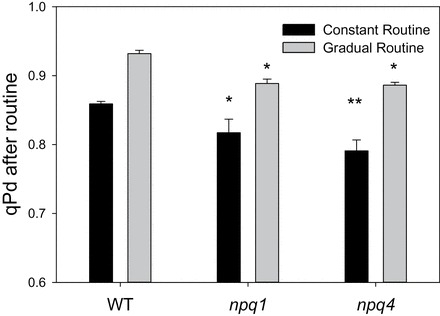
Comparison of maximum qPd for WT, *npq1*, and *npq4* plants measured under two different light regimes: constant high light (1500 μmol m^–2^ s^–1^) (black bars) and gradually increasing light (0, 90, 190, 280, 420, 625, 820, 1150, 1500 μmol m^–2^ s^–1^) (grey bars). Error bars represent the SEM (*n*=5 and 10 for constant and gradual routines, respectively). A single asterisk is used to signify this significant difference between mutants and WT plants, with a double asterisk representing a significant difference between *npq1* and *npq4* plants (*P*<0.05).

Figure 6 (grey bars) shows the degree of the photodamage (qPd parameter) at the end of the increasing actinic light illumination routine. qPd was decreased by 7% in WT plants compared with 11% for both *npq1* and *npq4*. This is evidence of a greater photoinhibition in the mutant plants. The difference was significant between the WT and mutants (ANOVA, *P*<0.01), but not between *npq1* and *npq4* (*t*-test, *P*=0.69). Plants are therefore more susceptible to RC damage when lacking the ability to form zeaxanthin (*t*-test, *P*=0.0038) or in the absence of PsbS (*t*-test, *P*=0.0012) compared with the WT.

**Fig. 7.**
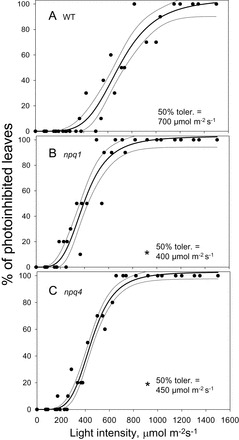
Relationship between the percentage of photoinhibited leaves and light intensity for (A) WT, (B) *npq1*, and (C) *npq4 Arabidopsis* plants. Data points are derivatives from Figs 2A–4A for WT, *npq1*, and *npq4*, respectively. Lines represent regression fit curves (sigmoidal, Hill, three parameter; *f*=*ax* ^b^/*c* ^b^+*x* ^b^) with 95% confidence values plotted using SigmaPlot12 (Systat Software, Inc., Chicago, IL, USA). A single asterisk is used to signify this significant difference between mutants and WT plants, with a double asterisk representing a significant difference between *npq1* and *npq4* plants (*P*<0.05).

The data shown in Figs 2A–4A were used to obtain light tolerance curves for the three genotypes (Fig. 7). This was obtained from the qPd values previously shown, by calculating the percentage of photoinhibited leaves as 100×*N*_rhombs_/*N*_total_, where *N*_rhombs_ represents the number of leaves photoinhibited (rhomboid symbols in Figs 2A–4A). The high variability in the protectiveness of NPQ between individual plants as well as leaves is evident. The maximum light intensity at which 100% of *npq1* and *npq4* leaves were still free from the signs of photoinhibition was ~200 μmol m^–2^ s^–1^. Fifty percent of leaves of the *npq1* and *npq4* mutants suffered photodamage at 400 μmol m^–2^ s^–1^ and 450 μmol m^–2^ s^–1^, respectively, although the regression curves were not significantly different between mutants (*z*-test, *P*<0.05). WT plants showed significantly better protection than both mutants throughout the routine (*z*-test, *P*>0.05), with 100% free from photoinhibition at ~400 μmol m^–2^ s^–1^, and 50% undamaged at 700 μmol m^–2^ s^–1^.

### Calculating the rates of NPQ formation and the onset of photoinhibition at constant high actinic light exposure

Upon constant illumination by 1500 μmol m^–2^ s^–1^ (Supplementary Fig. S1 at *JXB* online), a light intensity photoinhibitory for all three types of plants used in this study, pNPQ was unable to form fully as in the conditions of a gradually increased actinic light intensity (Figs 2–4). Hence, the onset of photoinhibition was found to be almost instant judging by the decline in qPd and PSII yield (Supplementary Fig. S3). Indeed, the sudden onset of photodamage resulted in a very different relationship between NPQ, qPd, and ΦPSII for all genotypes. In the leaves exposed to a constant high light, qPd declined particularly rapidly over the first three saturating pulses. This is in contrast to the gradual illumination routine where qPd was stable for the first four (*npq1* and *npq4*) or five (WT) pulses before rapidly declining. This behaviour is matched by the relationship between the measured and theoretical ΦPSII. Whilst in the gradual illumination routine the calculated and measured yield displayed a good match at somewhat lower light intensities, they immediately diverged in the constant high light illumination (Supplementary Fig. S3).

**Fig. 8.**
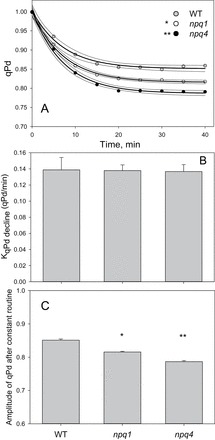
(A) Onset of qPd formation over a constant 1500 μmol m^–2^ s^–1^ light routine, illustrated in Fig. 1. Grey circles indicate WT, white *npq1*, and black *npq4 Arabidopsis* leaves. The darker lines indicate regression fit curves [exponential decay, single, three parameter; *f*=*y*_0_+*a*×exp(–*bx*)], and the lighter lines represent 95% confidence intervals, all plotted using SigmaPlot12 (Systat Software, Inc.). (B) Rate constant for qPd formation, obtained via the equation generated from the regression fit curve. Error bars show the SD. (C) Amplitude of qPd formation derived from the equation for the fitting of the regression curve (from A). Error bars illustrate the SD. A single asterisk is used to signify this significant difference between mutants and WT plants, with a double asterisk representing a significant difference between *npq1* and *npq4* plants (*P*<0.05).

The comparison between qPd values at the end of this new type of routine is displayed in Fig. 6 (black bars). It demonstrates that whilst under gradually increasing light exposure, *npq4* plants showed similar levels of photoinhibition to *npq1* plants (grey bars), they were affected more under a high light exposure routine, revealing a >20% decline in qPd by the end of illumination (black bars). Although qPd decline was not significantly altered at the end of the routine between *npq4* and *npq1* plants, it was significantly greater than that of the WT (*P*=0.011). Furthermore, ANOVA shows that the difference between all genotypes in qPd decline over the course of the constant routine was significant (*P*=6.49 × 10^−5^), with *t*-test analysis illustrating a significant difference between *npq1* and *npq4* plants (*P*=0.017). This vulnerability to a sudden high light exposure in *npq4* plants led to further investigation of the rates and amplitudes of qPd decline and NPQ formation. Figure 8A illustrates the onset of qPd decline during the constant high light routine. From the relative exponential decay regression lines, the rate constants of decay were obtained and plotted in Fig. 8B. A *z*-test showed there was no statistical difference between the genotypes in the rate of qPd decline over the routine (*P*>0.1). From the regression lines of Fig. 8A, the predicted amplitudes for qPd decline at the end of the procedure are shown in Fig. 8C. The predicted qPd decline is very similar to the measured results at the end of the constant routine (Fig. 6, black lines), thereby demonstrating the goodness of fit of the model. As was demonstrated in the measured qPd, the predicted qPd illustrated that *npq4* plants have significantly greater amplitude of photodamage than *npq1* (*P*<0.001) and WT plants (*P*<0.001), and that *npq1* plants are also less well protected than the WT (*P*<0.001).

**Fig. 9.**
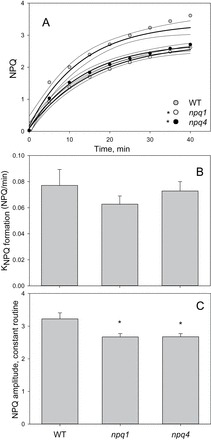
(A) Onset of NPQ formation over a constant 1500 μmol m^–2^ s^–1^ light routine, illustrated in Fig. 1. Grey circles indicate WT, white *npq1*, and black *npq4 Arabidopsis* leaves. The darker lines indicate regression fit curves {exponential rise to maximum, single, three parameter; *f*=*y*_0_+*a*[1–exp(–*bx*)]}, and the lighter lines represent 95% confidence intervals, all plotted using SigmaPlot12 (Systat Software, Inc.). (B) Rate constant for NPQ formation, obtained via the equation generated from the regression fit curve. Error bars show the standard deviation. (C) Amplitude of NPQ formation derived from the equation for the fitting of the regression curve. Error bars illustrate the SD. A single asterisk is used to signify this significant difference between mutants and WT plants, with a double asterisk representing a significant difference between *npq1* and *npq4* plants (*P*<0.05).

The analysis of NPQ kinetics presented in Fig. 9 illustrates that WT plants form NPQ at a quicker rate and to a significantly greater amplitude (*P*<0.01) than both mutants. Interestingly, whilst the total qPd decline was higher in the *npq4* mutant, no difference was found under constant high light routine in the measured and predicted NPQ parameters between the two mutants. It was hypothesized that this difference was due to a strong photoinhibitory qI component in the *npq4* mutant. In this scenario, the amount of pNPQ formed in *npq4* plants was expected to be smaller and hence less efficient than that in *npq1* and the WT. It was therefore decided to test this possibility by calculating the amount of qI contributing to the total NPQ for the three genotypes.

### Estimation of qI using the uncoupler nigericin

**Fig. 10.**
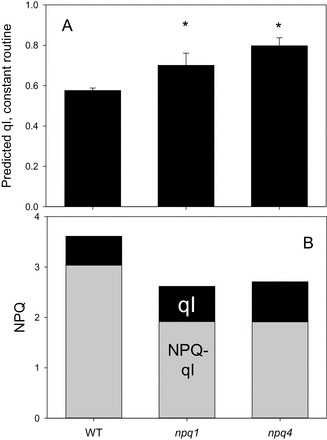
(A) Amplitude of qI for WT, *npq1*, and *npq4* leaves. This was derived from the relationship between NPQ and qPd in nigericin-infiltrated leaves (see text and Supplementary Fig. S4 at *JXB* online for more detail). Error bars represent the SEM (*n*=5). (B) Proportions of relative amplitude of photoinhibitory NPQ (qI) and non-photoinhibitory NPQ (NPQ–qI) for WT, *npq1*, and *npq4 Arabidopsis* plants. See in text for details. A single asterisk is used to signify this significant difference between mutants and WT plants, with a double asterisk representing a significant difference between *npq1* and *npq4* plants (*P*<0.05).

In order to test the above hypothesis, the constant 1500 μmol m^–2^ s^–1^ illumination procedure was applied to leaves infiltrated with nigericin. Since nigericin inhibits the rapidly and slowly reversible components of NPQ related to photoprotection (qE and qZ), only the qI component should remain after this treatment. Therefore, in leaves infiltrated with nigericin, NPQ is equal to qI. After performing the infiltration, leaves were subjected to the constant light routine. From this, the relationship between NPQ (here it is qI) and qPd can be presented (Supplementary Fig. S4A at *JXB* online). Leaves from different genotypes showed a practically identical trend. Dashed lines on Supplementary Fig. S4A highlight the relationship between the values of qPd obtained at the end of the continuous high light procedure without nigericin (Figs 6, 8A) and qI. This method allowed estimation of the amount of the qI component in NPQ measured at the end of the procedure. Figure 10A shows the derived qI for all three genotypes (Fig. 10A). The total NPQ measured at the end of the routine (Fig. 9A) was then separated into qI and (NPQ–qI) (Fig. 10B). Figure 10 illustrates that *npq4* plants have the highest qI, then *npq1*, and the WT has the least. These results were significantly different between WT and mutant plants (*z*-test, *P*<0.05 and 0.001 for *npq1* and *npq4*, respectively) and provide an explanation as to why the two mutants form a similar amount of NPQ, while qPd is different between them under constant high light (Fig. 8); this is because total NPQ also accounts for qI (Fig. 10). In order to confirm the results obtained from the routine performed on nigericin-infiltrated leaves, a modified constant high light routine was applied to whole intact leaves (Supplementary Fig. S4B). After the constant eight step 1500 μmol m^–2^ s^–1^ routine application, the measuring light remained on for 1h with an SP being applied every 10min. Only the qI component of NPQ will remain after this time (Adams *et al.*, 2006; Jahns and Holzwarth, 2012; Ruban *et al.*, 2012), therefore another estimation of this relationship can be calculated. The results obtained from this support the results obtained from the nigericin infiltration experiment, with *npq4* leaves having the most qI, then *npq1*, and then the WT, with both mutants having significantly more than the WT leaves (*z*-test; *P*<0.05 and 0.001 for *npq1* and *npq4*, respectively). Plants with both PsbS and zeaxanthin therefore have less qI than either of the mutants. *npq1* mutants had less photodamaged RCIIs than *npq4*. Figure 10B demonstrates that the reason total NPQ was higher in *npq4* plants compared with *npq1* (in Fig. 9A) was due to higher qI. (NPQ–qI) was greatest in the WT, then *npq1*, and was lowest in *npq4*. This result supports the data shown in Fig. 8 which show that qPd decline is the greatest in *npq4* mutants. This reflects the greater vulnerability of these plants to photodamage than *npq1* plants when suddenly exposed to strong light.

## Discussion

The aim of this research was to assess quantitatively and compare the contribution of the PsbS protein and the pigment zeaxanthin to the protective efficiency of NPQ. Continuing on from the theory developed by Ruban and Murchie (2012) and the established routine from Ruban and Belgio (2014), the protectiveness of NPQ in WT, *npq1*, and *npq4* plants was effectively calculated based upon the use of a non-invasive fluorescence approach. First, a gradually increasing light routine was applied to represent sunrise over a field on a cloudless day (Fig. 1). Here plants are allowed to form maximum pNPQ without drastic fluctuations in light intensity. Therefore, the selection pressure on the plant was only the total amount of NPQ it can form, not the rate at which it accommodates the changing light environment (Figs 2A–4A). It has previously been demonstrated that *npq1* (Niyogi *et al.*, 1997) and *npq4* (Johnson and Ruban, 2011) plants can form qE, albeit with different kinetic properties. Furthermore, Külheim and Jansson produced two papers comparing a number of physiological characteristics between *npq1* and *npq4* mutant plants (Külheim *et al.*, 2002; Külheim and Jansson, 2005). Grown under similar constant light conditions to those employed in this project, the group found that *F*_v_/*F*_m_, rosette width (during the vegetative growth stages), photosynthetic pigment composition, seed and fruit production, and anthocyanin accumulation did not differ between the two mutants. This research has improved on these findings and offered a novel insight into how much NPQ can be formed in *npq1* and *npq4* plants, and how big its protective component is. It was revealed that there was no significant difference (*P*=0.77) between the total amount of NPQ formed in the mutants, thereby showing that under gradually increasing light conditions it is not preferable to have either zeaxanthin or PsbS, but both are required to form maximum NPQ. Assessing pNPQ in the mutants allowed the maximum light intensity (when 100% of leaves have qPd >0.98) tolerated to be compared (Fig. 5). Both *npq1* and *npq4* plants were able to tolerate the same maximum light intensity without RCII damage, and a similar amount of light caused photoinhibition in 50% of studied leaves (Fig. 7). Although WT plants developed more NPQ and pNPQ, the relationship between pNPQ and light intensity was very similar for all three genotypes. This suggests that only the absolute amount of pNPQ matters for photoprotection regardless of the PsbS or zeaxanthin composition of the photosynthetic membrane. This conclusion was similar to the findings of Ruban and Belgio (2014) who used plants with only varying amounts of PsbS protein. The results also confirm the important role of the contribution and efficiency of NPQ in the protection of PSII against photodamage, a conclusion which differs somewhat from the findings or Sarvikas *et al*. (2006).

The second experimental approach consisted of the sudden illumination of plants with constant high light. This is an extreme case of rapid light fluctuations occurring, for instance, upon sunlight breaking through a cloud or canopy (Supplementary Fig. S1 at *JXB* online). An intensity of 1500 μmol m^–2^ s^–1^ was chosen as the constant high light intensity, because the gradual routine showed that no leaves could survive under this light intensity without suffering photoinhibition. When high light is provided from the start, the rate of NPQ formation was shown to be imperative since *npq4* plants suffered more than *npq1* and WT plants (Fig. 6, black bars). It has been previously established that *npq4* plants form slower NPQ over the first 30–60min compared with the WT (Horton *et al.*, 2000; Li *et al.*, 2000; Johnson and Ruban, 2010) as PsbS promotes the membrane re-organization required for the rapid establishment of the NPQ state (Goral *et al.*, 2012). It was found that the reason why the rates of NPQ formation were not significantly different across the three genotypes was due to a greater contribution of qI to NPQ in *npq4* plants (Figs 9, 10). This was in agreement with the fact that the qPd decline at the end of the routine was significantly greater in *npq4* plants (Fig. 8C).

The two routines were developed to mimic two types of contrasting natural environment, but the stepwise increase in actinic light intensity is a current instrumental limitation. Tuning the distance between the light emitter and fibre-optics was an attempt to increase the range of light intensities in order to create a greater resolution in the light tolerance curves (Fig. 7). However, future developments in the proposed method will be aimed at improving its sensitivity. The brief dark periods between different actinic illuminations will still be required to measure calculated and actual *F*_o_’ to determine the onset of photoinhibition. This brief 10 s interruption during the light treatment is a quicker, less disruptive, and more reliable way of measuring photoinhibition (as qPd), rather than allowing qE relaxation over ~5min and then calculating the remaining, slowly reversible NPQ (Maxwell and Johnson, 2000) (for an extended discussion, see Ruban and Murchie, 2012). By combining the results from the two routines, it has been possible to determine that WT plants form fast and relatively large NPQ, which protects more RCs than the fast, but smaller NPQ in *npq1*, and the slower and reduced NPQ in *npq4* plants. Under gradually increasing light intensities there was consequently no discernible difference between photoprotectiveness in *npq1* and *npq4* plants (Figs 5, 7), but, at high light, *npq1* plants are better protected by simply being able to adjust more quickly to the rapid onset of strong photoinhibitory light (Figs 6, 8).

## Supplementary data

Supplementary data are available at *JXB* online.

Figure S1. Scheme of induction of chlorophyll fluorescence from an *npq4* plant with an eight step constant 1500 μmol m^–2^ s^–1^ light routine.

Figure S2. Relationship between maximum protective capacity and light intensity during a gradually increasing routine.

Figure S3. Relationship between PSII yield, qPd, and NPQ under a constant 1500 μmol m^–2^ s^–1^ light routine on WT, *npq1*, and *npq4 Arabidopsis* intact leaves.

Figure S4. (A) Relationship between photoinhibitory NPQ (qI) and qPd for detached leaves infiltrated with nigericin illuminated with a constant 1500 μmol m^–2^ s^–1^ light routine. (B) Depicts the routine described in Fig. S1; however, after the routine had finished, the measuring light remained on for 1h, with an SP being applied every 10min.

Supplementary Data
