# Supplementary material for: Comparison of the protective effectiveness of NPQ in Arabidopsis plants deficient in PsbS protein and zeaxanthin
Source: J Exp Bot. 2014 Nov 26;66(5):1259–70. doi: 10.1093/jxb/eru477 (PMC4339590; doi:10.1093/jxb/eru477)
Supplement: Supplementary Data [file supp_66_5_1259__index.html]

Comparison of the protective effectiveness of NPQ in Arabidopsis plants deficient in PsbS protein and zeaxanthin — Comparison of the protective effectiveness of NPQ in Arabidopsis plants deficient in PsbS protein and zeaxanthin — Supplementary Data 

# Comparison of the protective effectiveness of NPQ in *Arabidopsis* plants deficient in PsbS protein and zeaxanthin

## Supplementary Data

Data files

**Files in this Data Supplement:**

- Supplementary Data - Supplementary Data
