# Supplementary material for: Comparison of the protective effectiveness of NPQ in Arabidopsis plants deficient in PsbS protein and zeaxanthin
Source: J Exp Bot. 2014 Nov 26;66(5):1259–70. doi: 10.1093/jxb/eru477 (PMC4339590; doi:10.1093/jxb/eru477)
Supplement: Supplementary Data [file supp_eru477_jexbot135582_file001.pdf]

Comparison of the protective effectiveness of NPQ in *Arabidopsis* plants deficient in PsbS protein and zeaxanthin.

Maxwell A. Ware, Erica Belgio and Alexander V. Ruban[#]

School of Biological and Chemical Sciences, Queen Mary University of London, Mile End Road, London E1 4NS, UK

[#]Correspondence: a.ruban@qmul.ac.uk

Supporting information

Supplemental Figure 1 - Ware *et al.*

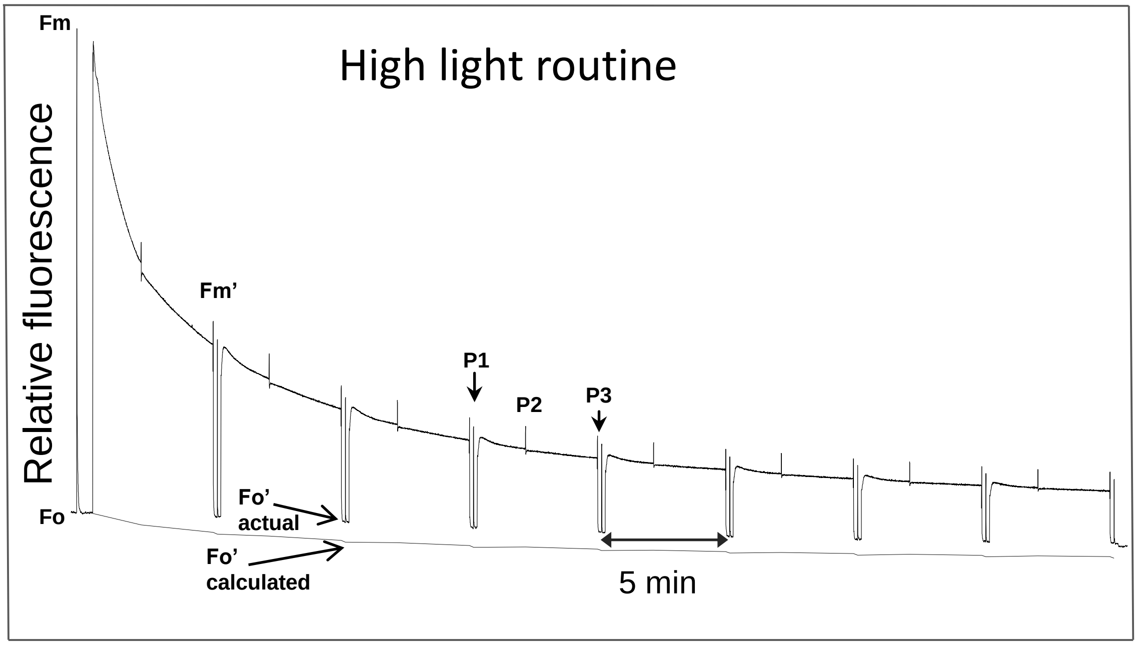

Figure S1 Scheme of induction of chlorophyll fluorescence from an *npq4* plant with eight step constant 1500 µmol m^{-2} s^{-1} light routine. For detailed explanation of routine development see Ruban & Belgio (2014). P1 illustrates the saturating pulse (SP) before actinic light (AL), P2 during AL, and P3 after AL and far red (FR). All parameters measured were done so at P1. The difference between actual and calculated Fo' was used to calculate qPd. See 'Materials and methods' for a detailed description. The timing scheme of the qPd calculation and darkness step of the routine is: (AL off)(FR on)-(7 s)-(SP)-(5 s)-(AL on/FR off).

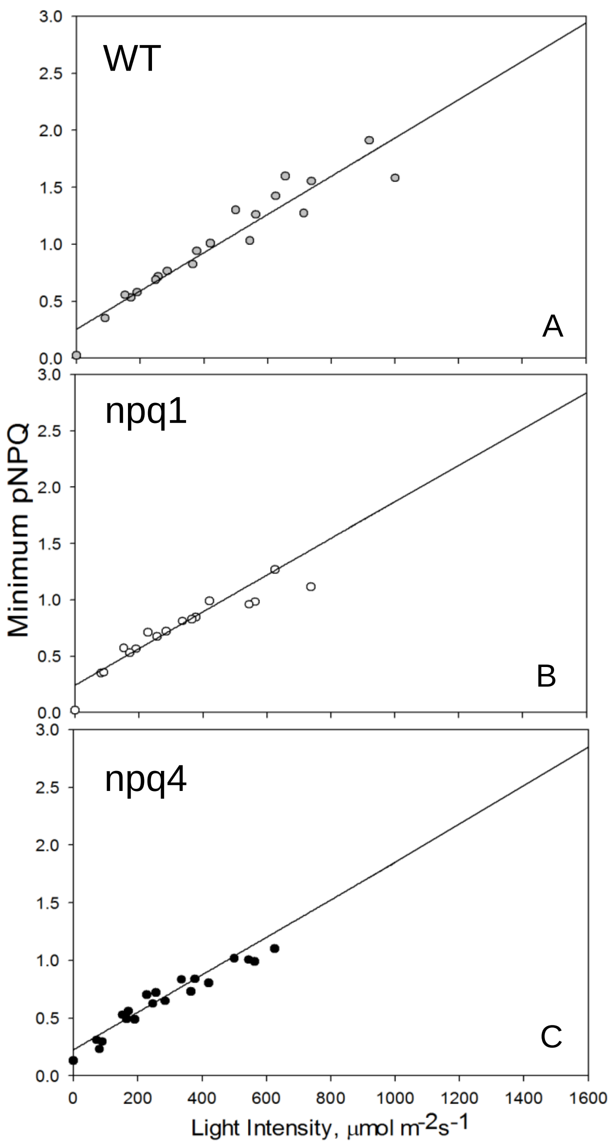

Figure S2 Relationship between maximum protective capacity and light intensity during a gradually increasing routine (see Fig. 1 and in text description). The straight line (Standard Curves, Linear Curve; f = y0+a*x) was plotted using the regression analysis on SigmaPlot12 (Systat Software, Inc., Chicago, USA). Data points were taken from Fig. 5 representing the lowest NPQ value corresponding to qPd > 0.98 at a given light intensity for **A** WT (f = 0.2566+0.0017*x) **B** *npq1* (f = 0.2437+0.0016*x) **C** *npq4* (f = 0.2267+0.0016*x). The gradient can therefore be used to estimate the minimum NPQ needed for 100% RC protection at each actinic light intensity.

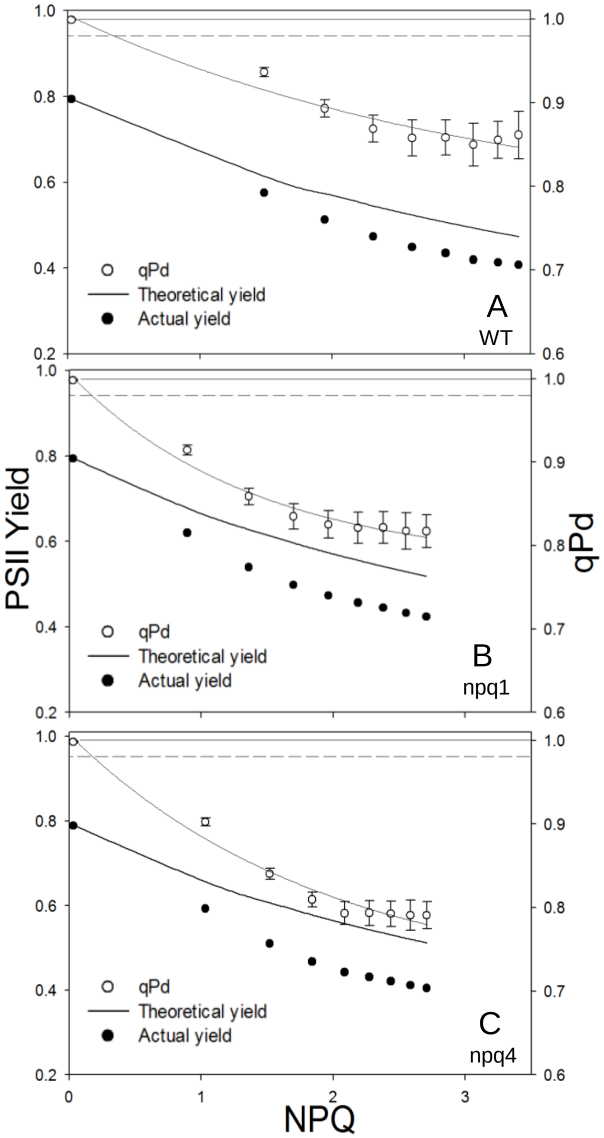

Figure S3 Relationship between PSII yield, qPd and NPQ under a constant 1500 µmol m^{-2} s^{-1} light routine on **A** WT, **B** *npq1* and **C** *npq4 Arabidopsis* intact leaves. The figure also explains the actual and theoretical relationship between NPQ and ΦPSII. The equation used to describe this relationship is explained in the methods. Error bars illustrate SEM (n = 5).

Supplemental Figure 4 - Ware *et al.*

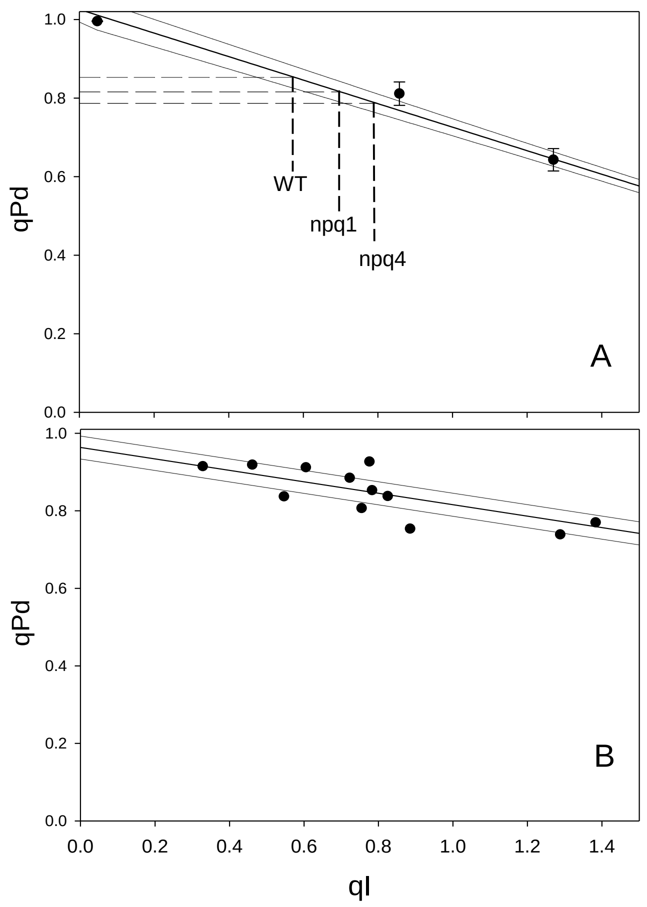

Figure S4 A Relationship between photoinhibitory NPQ (qI) and qPd for detached leaves infiltrated with nigericin illuminated with a constant 1500 µmol m^{-2} s^{-1} light routine. Extrapolation lines are included for all genotypes to show the relationship between qPd and qI. Error bars represent the SEM (n = 5). The regression line (Standard Curves, Linear Curve; f = y0+a*x) was plotted using SigmaPlot12 (Systat Software, Inc., Chicago, USA). **B** Depicts the routine described in Fig. S1; however, after the routine had finished the measuring light remained on for 1 hr, with an SP being applied every 10 min. The final qPd and NPQ values recorded for 14 separate whole intact leaf measurements were plotted. The regression line and 95% confidence interval (Standard Curves, Linear Curve; f = y0+a*x) were plotted using SigmaPlot12 (Systat Software, Inc., Chicago, USA).
